# Acid ground nano‐realgar processed product inhibits breast cancer by inducing mitophagy via the p53/BNIP3/NIX pathway

**DOI:** 10.1111/jcmm.17917

**Published:** 2023-08-23

**Authors:** Jiahui Fang, Xue Zou, Ling Gong, Juan Xi, Yi Liu, Xiaoli Yang, Xiuqiao Zhang, Chun Gui

**Affiliations:** ^1^ College of Pharmacy Hubei University of Chinese Medicine Wuhan Hubei China; ^2^ Department of Pharmacy, Taihe Hospital Hubei University of Medicine Shiyan Hubei China; ^3^ College of Inspection Hubei University of Chinese Medicine Wuhan Hubei China

**Keywords:** acid ground nano‐realgar processed product, breast cancer, MDA‐MB‐435S cell, mitophagy, p53/BNIP3/NIX

## Abstract

Breast cancer is a highly prevalent malignancy with the first morbidity and the primary reason for female cancer‐related deaths worldwide. Acid ground nano‐realgar processed product (NRPP) could inhibit breast cancer cell proliferation and induce autophagy in our previous research; however, the underlying mechanisms are still unclear. Therefore, this research aimed to verify whether NRPP induces breast cancer mitophagy and explore the mitophagy‐mediated mechanism. Primarily, rhodamine‐123 assay and transmission electron microscopy were applied to detect mitochondrial membrane potential (MMP) and ultrastructural changes in the MDA‐MB‐435S cells, respectively. Mito‐Tracker Green/Lyso‐Tracker Red staining, western blot, immunofluorescence and RT‐PCR were used to explore molecular mechanisms of NRPP‐induced mitophagy in vitro. MDA‐MB‐435S breast cancer xenograft models were established to assess the activity and mechanisms of NRPP in vivo. Our results showed that NRPP decreased MMP and increased autophagosome numbers in MDA‐MB‐435S cells and activated mitophagy. Furthermore, mitophagy was consolidated because mitochondria and lysosomes colocalized phenomenology were observed, and the expression of LC3II/I and COXIV was upregulated. Additionally, we found the p53/BNIP3/NIX pathway was activated. Finally, NRPP inhibited tumour growth and downregulated the levels of TNF‐α, IL‐1β and IL‐6. Necrosis, damaged mitochondria and autophagosomes were observed in xenograft tumour cells, and proteins and mRNA levels of LC3, p53, BNIP3 and NIX were increased. Overall, NRPP inhibited MDA‐MB‐435S cell proliferation and tumour growth by inducing mitophagy via the p53/BNIP3/NIX pathway. Thus, NRPP is a promising candidate for breast cancer treatment.

## 1 INTRODUCTION

According to the global cancer statistics in 2020, 2,261,419 new cases and 684,996 new deaths because of breast cancer were recorded in 185 countries around the world. Breast cancer remains a highly prevalent malignancy, surpassing lung cancer and ranking first in the world.^1^ Furthermore, it is the main reason for female cancer‐related deaths all over the world. Traditional therapies, including surgery, radiotherapy, chemotherapy and endocrine therapy, have many inescapable restrictions,^2^, ^3^ unpleasant side effects, bad prognosis and worse survival call for the development of original and promising approach for breast cancer therapies.^4^, ^5^, ^6^

In the past few years, traditional Chinese medicine (TCM) developed rapidly, and many studies have reported its antitumour effects via multiple mechanisms with minimal side effects.^7^, ^8^ Arsenic sulphide (As_4_S_4_), namely realgar, is a mineral TCM with a pronounced therapeutic effect in leukaemia^9^, ^10^ and an excellent antineoplastic effect.^11^, ^12^ However, its high toxicity, low solubility and poor bioavailability hinder the clinical applications.^13^, ^14^ Nano‐preparations made from inorganic compounds such as copper oxide,^15^ zinc oxide^16^ and realgar,^17^ have already been researched the antineoplastic activities. Given this, we prepared realgar nanoparticles with enhancing water solubility in our previous study. To reduce the toxicity of leached arsenic trioxide, we processed realgar nanoparticles with levigating grinded ball‐milled realgar in 5% hydrochloric acid solution and acquired acid ground nano‐realgar processed product (NRPP), which had a better antitumour effect than crude realgar.^18^ Furthermore, we found that NRPP could suppress the growth of MCF‐7, MDA‐MB‐231 and MDA‐MB‐435S human breast cancer cell lines. The half maximal inhibitory concentration (IC_50_) of MDA‐MB‐435S cells NRPP‐treated was 65.78 μg/mL at 12 h. Meanwhile, we found NRPP may induce autophagy by acting on mitochondria in MDA‐MB‐435S cells.^19^ However, the molecular mechanisms of NRPP‐induced autophagy remain unclear.

Mitochondria are involved in cell biosynthesis control, energy generation and signal transduction. They can be damaged by nutritional deficiency, hypoxia, DNA damage and inflammation, interrupting cell energy supply and leading to cell death.^20^, ^21^ Mitophagy is a kind of selective autophagy that can distinctively remove aged and dysfunctional mitochondria to sustain intracellular homeostasis and metabolism and is essential for mitochondria quality control.^22^ It is participate in the regulation of lung, breast, liver and stomach cancers.^23^, ^24^ Moreover, mitophagy can reduce the resistance and strengthen tumour cell sensitivity to chemotherapy drugs.^25^, ^26^ Thus, mitophagy is a main character in the occurrence, deterioration and cure of cancer, and it may be a prospective therapeutic target.

Therefore, the purpose of our study was to evaluate the anti‐breast cancer effect of NRPP and investigated the unknown mechanisms, focusing on the signalling pathway of NRPP‐activated mitophagy in vitro and in vivo. Our research might provide insight into developing and offering a theoretical foundation for new breast cancer treatments based on realgar nanoparticles with specific targets.

## 2 MATERIALS AND METHODS

### 2.1 Chemicals

NRPP was previously prepared in our laboratory.^18^ Crude realgar was purchased from Shimen, Hunan Province, ground and sieved with a 200‐mesh to gain realgar medicinal powder. The realgar powder was ball milled using a planetary high‐energy ball mill (Shanghai, China) (parameters: rotation speed, 400 r/min; ball‐material ratio, 40:1; grinding aid 10 mL of water and ball‐milling time, 16 h). Then, we got raw realgar nanoparticles with 157.3 nm particles. The raw realgar nanoparticles were repeatedly ground with 5% hydrochloric acid (#C937419, ≥98%; Sinopharm, Shanghai, China) and kept for 12 h. Next, suction filtered and vacuum dried. Then, NRPP was got. The average particle size of the NRPP was 137.7 ± 4.5 nm. Cisplatin (DDP, ≥98%) was obtained from Yuanye Biology Co. Ltd., China.

Primary antibodies against GAPDH (#8884S) were bought from CST (Danvers, MA, USA). LC3 (#14600‐1‐AP), COXIV (#11242‐1‐AP), Parkin (#14060‐1‐AP), PINK1 (#23274‐1‐AP) and goat anti‐rabbit second antibody (#SA00001‐2) were obtained from Proteintech (Chicago, IL, USA). BNIP3 (#A5683), NIX/BNIP3L (#A6283) and p53 (#A11232) were purchased from Abclonal (Wuhan, China). Carboxymethylcellulose sodium (CMC‐Na, #20190304) was purchased from Sinopharm Group (Shanghai, China). Mito‐Tracker Green (MTG, #C1046) and Lyso‐Tracker Red (LTR, #C1048) were bought from Beyotime (Shanghai, China).

### 2.2 Cell culture

Human breast cancer MDA‐MB‐435S cells were obtained from the Cell Center of the Chinese Academy of Medical Sciences (#CL‐0151) and were cultured in Dulbecco's Modified Eagle's Medium (DMEM, #AC10253739; HyClone) containing 10% foetal bovine serum (FBS, #42F3495K; Gibco) and 1% penicillin/streptomycin (#J180014; HyClone) at 37°C in a humidified 5% CO_2_ environment.

### 2.3 Rhodamine 123 Assay (Rh‐123)

Rh‐123 (#C2007; Beyotime) was employed to test mitochondrial membrane potential (MMP). Cells were seeded in 6‐well plates for 24 h, were collected after exposure to NRPP (0, 20, 40 and 80 μg/mL) for 9 h and stained with 1 μg/mL Rh‐123 in a dark room for 30 min. All samples were tested by flow cytometry (FCM, BD FACSCalibur, USA).

### 2.4 Transmission electron microscopy (TEM)

MDA‐MB‐435S cells were treated with 80 μg/mL NRPP for 9 h. The harvested cells or a 2 mm section of tumour tissue were fixed in 2.5% phosphate‐buffered glutaraldehyde overnight. After washing twice with phosphate buffer (PBS, #AC11496277; HyClone) and fixing with 1% OsO_4_ buffer, the cells were dehydrated by ethanol at a gradient concentration for 15 min each time and embedded in acetone. Next, cells were cut into ultrathin slices and stained with a 2% uranium acetate‐saturated aqueous solution and lead citrate. Images were obtained by an HT7800 TEM (Hitachi, Japan).

### 2.5 Colocalization of mitochondria with lysosome

MTG and LTR fluorescent probes were applied to detect the colocalization of mitochondria with lysosome. Cells were incubated in polylysine‐coated confocal cuvettes for 24 h and exposed to NRPP (0, 20, 40 and 80 μg/mL) for 9 h. The cells were cultured with 400 nM MTG and 400 nM LTR dyes after washing with PBS, dyed for 40 min, washed with serum‐free DMEM and photographed by a laser confocal microscope (FV3000; Olympus).

### 2.6 Western blot analysis

MDA‐MB‐435S cells were treated with NRPP (0, 20, 40 and 80 μg/mL) for 9 h or 40 μg/mL NRPP for different durations (0, 3, 6 and 9 h). Total protein was isolated by centrifuging the fully lysed cell homogenate at 4°C at 13523 *g*, containing 250 μL lysis buffer (#P0013; Beyotime) and 1% phenylmethyl sulfonyl fluoride (Beyotime). Mitochondrial or cytosolic protein was operated by the Cell Mitochondria Isolation Kit (#C3601; Beyotime). The samples were electro‐transferred onto polyvinylidene fluoride membranes (PVDF; Millipore) after separation and closed in 5% nonfat milk for 90 min. PVDF membranes were then incubated with different primary antibodies overnight and secondary antibodies for 1 h. Finally, protein expression was detected by enhanced chemiluminescence (#MA0186, ECL; Meilunbio) in a Gene system (Syngene) and quantified using the ImageJ software.

### 2.7 Immunofluorescence analysis

MDA‐MB‐435S cells were incubated in polylysine‐coated confocal cuvettes for 24 h and then exposed to NRPP (0, 20, 40 and 80 μg/mL) for 9 h. Then, cells were dyed with DMEM containing 400 nM MTG for 40 min and closed with 1% bovine serum albumin (BSA, #735094; Amresco) in PBS for 1 h. After incubation with primary antibody, Cy3‐labelled goat anti‐rabbit (#A0516; Beyotime) secondary antibody was added in cells at a dilution of 1:500 with 3% BSA in PBS buffer for 90 min. Nuclei were dyed with 5 μg/mL DAPI (#C1002; Beyotime) for 3 min at RT. Finally, immunofluorescence results were taken by the laser confocal microscope.

### 2.8 Quantitative RT‐PCR

Total RNA from MDA‐MB‐435S cells or tumour tissues were extracted by the RNA prep Pure Cell Kit (#DP430; Tiangen) and RNA prep Pure Tissue Kit (#AKG0730A; Tiangen), respectively. Reverse transcription to cDNA was performed by the PrimeScriptTM RT reagent Kit (#AK81976A; Takara). RT‐PCR was tested on a StepOnePlus System (Applied Biosystems) using TB Green® Premix Ex TaqTM II (Takara). The results were analysed by 2^−ΔΔCT^ way. The following are primer sequences in Table 1.

**TABLE 1:** Sequences of primers.

Gene	Forward primer (5′–3′)	Reverse primer (5′–3′)
Human LC3B	CTTCTATGCCATTGACTGTGCTGTTG	CACGGATGCTCCACTCTCATTCTTC
Mouse LC3B	GCAGGGTAAACGGGCTGTGTG	GAGTGAGGACTTTGGGTGTGGTTC
Human p53	GAAGGCTGTCAGTCGTGGAAGTG	GCAAGAGGCAGAAATGTAAATGTGGAG
Mouse p53	GATTGGCTGGCTGTGACTGTCTC	GCACTTACTTCCTCCTGGCTGAATC
Human BNIP3	CACCTCTGCCTGTCCGATTTCAC	GAGAGTGCTTGCTGCTTCCATCC
Mouse BNIP3	TTAAAGGGTGCGTGCGGGTTATC	GGTGGACAGCAAGGCGAGAATC
Human NIX	TGAACAGCAGCAATGGCAATGATAATG	TGTGGATGGAGGATGAGGATGGTAC
Mouse NIX	GGCTCGGCATCTATATTGGA	AAATGCCCCAACAGAGTTTG
Human GAPDH	CAGGAGGCATTGCTGATGAT	GAAGGCTGGGGCTCATTT
Mouse GAPDH	GTTTGTGATGGGTGTGAACC	TCTTCTGAGTGGCAGTGATG

### 2.9 MDA‐MB‐435S breast cancer xenograft models

Female BALB/c nude mice were obtained from Changzhou Cavens Experimental Animal Co. Ltd. (Jiangsu, China, certificate no. SCXK 2016‐0010). The mice were housed in a sterile environment at 25 ± 2°C, with a 12 h light/dark cycle and adequate water and food. Every mice‐related procedure was gained agreement for the Institutional Animal Care and Use Committee of the Hubei University of Chinese Medicine.

MDA‐MB‐435S cells (1 × 10^7^/mouse) were resuspended in PBS and subcutaneously injected into the right flank of the mice rapidly. Tumorigenicity of the cells was monitored for 2 weeks. When the tumour volume was approximately 150 mm^3^, all mice were split at random into four groups (eight mice/group). The control mice were orally administered 0.4 mL 0.5% CMC‐Na solution once every alternate day. Mice in low‐dose NRPP (NRPP‐L) and high‐dose NRPP (NRPP‐H) groups were orally administered NRPP at 250 mg/kg and 500 mg/kg once every other day. DDP(#B12J10L97601, 2 mg/kg), the positive control, was diluted in saline and orally administered every 3 days. Measuring mice weight and tumour volume every 5 days. The tumour volume was calculated as: length × width^2^/2.

On Day 30, collecting blood samples and centrifuged at 845 *g*. Serum samples were used for ELISA analysis. The tumours and organ tissues were weighed and photographed. Parts of the tumour tissues were cut into paraffin sections for TEM, haematoxylin and eosin and immunohistochemistry (IHC). Parts of the tumour tissues were fixed in Nucleic Acid and Protein and Stabilization Reagent (#R0121, Beyotime) for western blot and RT‐PCR. The rest were frozen in liquid nitrogen.

### 2.10 ELISA assay

The levels of TNF‐α, IL‐6 and IL‐1β in the mouse serum were detected by ELISA kits (#2411761618, #1321753618 and #1161759618; BOSTER). All procedures were performed as per the manufacturer's instructions.

### 2.11 Haematoxylin and eosin staining

The tumour tissues were dehydrated, embedded in paraffin (Amresco) and chop in 4 mm‐thick slices, which were dewaxed, rehydrated, dyed with haematoxylin and eosin (Solarbio) and sealed with neutral gum. Photographs were obtained by observing tumour morphology under a microscope (Olympus).

### 2.12 IHC staining

Tumour tissue sections were dewaxed, rehydrated and treated with hydrogen peroxide. The sections were closed with BSA and then reacted with primary antibodies for the night and HRP‐conjugated antibodies for 1 h. Haematoxylin was used for counterstaining the tissues after the peroxidase reaction with diaminobenzidine (DAB, #K5007; DAKO). The sections were gradient concentrations dehydrated by ethanol in gradient concentrations and blocked with neutral gum. Photographs were captured under a microscope. Positive dying was indicated by brown regions. Image Pro Plus software (version 6.0) was used to quantitatively analyse the mean optical density of positive expression.

### 2.13 Statistical analysis

Data are presented as means ± SD. Multiple comparisons were analysed by one‐way anova with SPSS software (version 22.0).

## 3 RESULTS

### 3.1 NRPP damaged mitochondria and decreased MMP in MDA‐MB‐435S Cells

**FIGURE 1:**
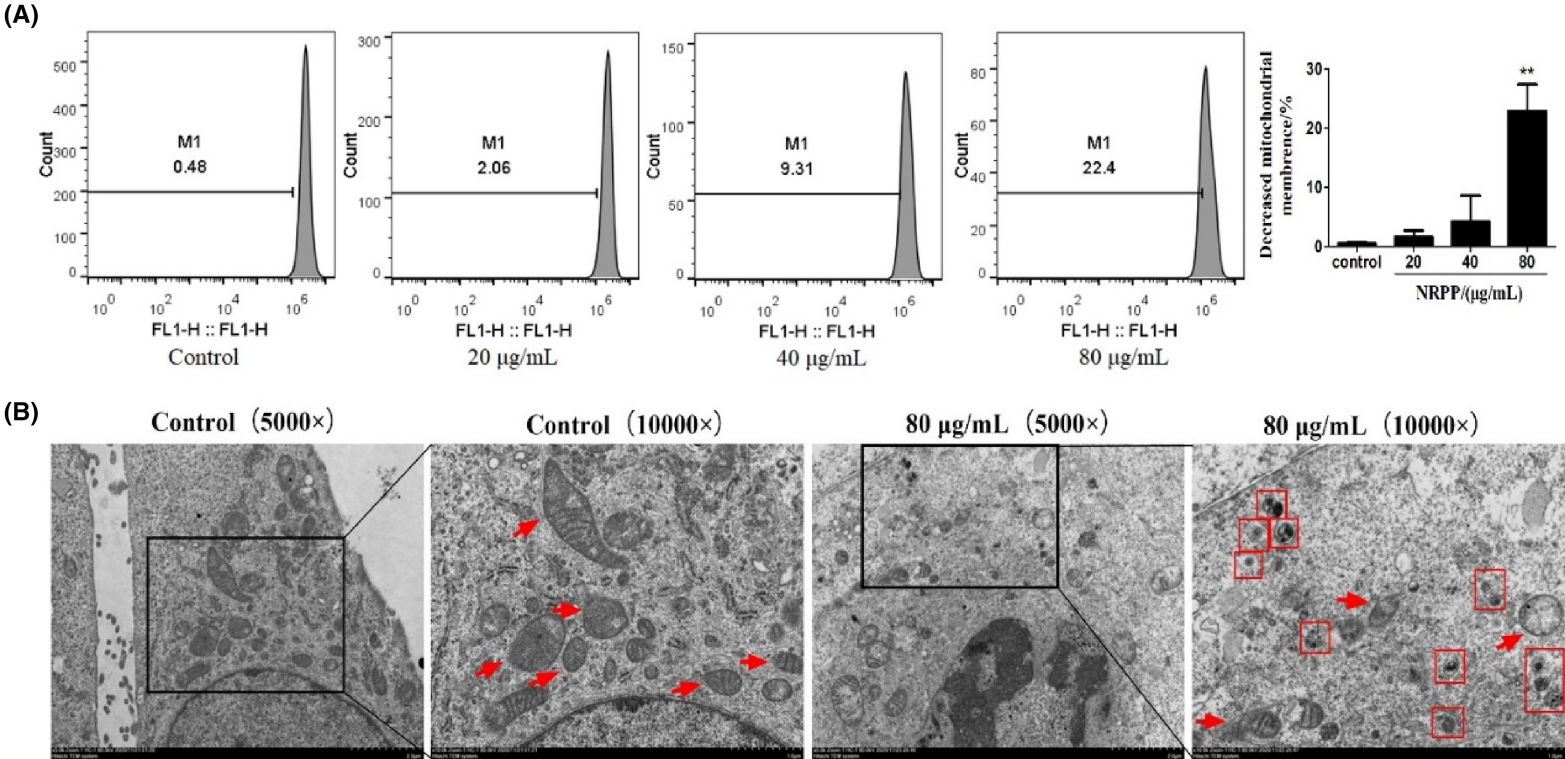
NRPP decreased MMP and induced autophagy in MDA‐MB‐435S cells. The cells were exposed to different concentrations of NRPP for 9 h. (A) MMP was tested by Rh‐123 assay via FCM. (B) The cellular ultrastructure was observed under TEM. The magnifications of the images were 5000× and 10,000×. Red arrows: mitochondria; red squares: autophagosomes. **p < 0.01 vs. control group.

The cells were stained with Rh‐123 after treatment with 0, 20, 40 or 80 μg/mL NRPP, and the changes in MMP were tested by FCM. The results showed that with an increase in NRPP concentration, the peak shifted to the left and the fluorescence intensity decreased (Figure 1A), indicating that the MMP decreased gradually.

Morphological changes were observed under TEM after NRPP‐treated with 80 μg/mL (Figure 1B). In the control, mitochondria were normal, with a clear and complete cristae structure. But in the NRPP group, the structure of mitochondria was destroyed, mitochondrial cristae were broken, and autophagosomes appeared in the cells. Therefore, mitophagy might be activated by NRPP in MDA‐MB‐435S cells.

### 3.2 NRPP‐induced mitophagy in MDA‐MB‐435S Cells

**FIGURE 2:**
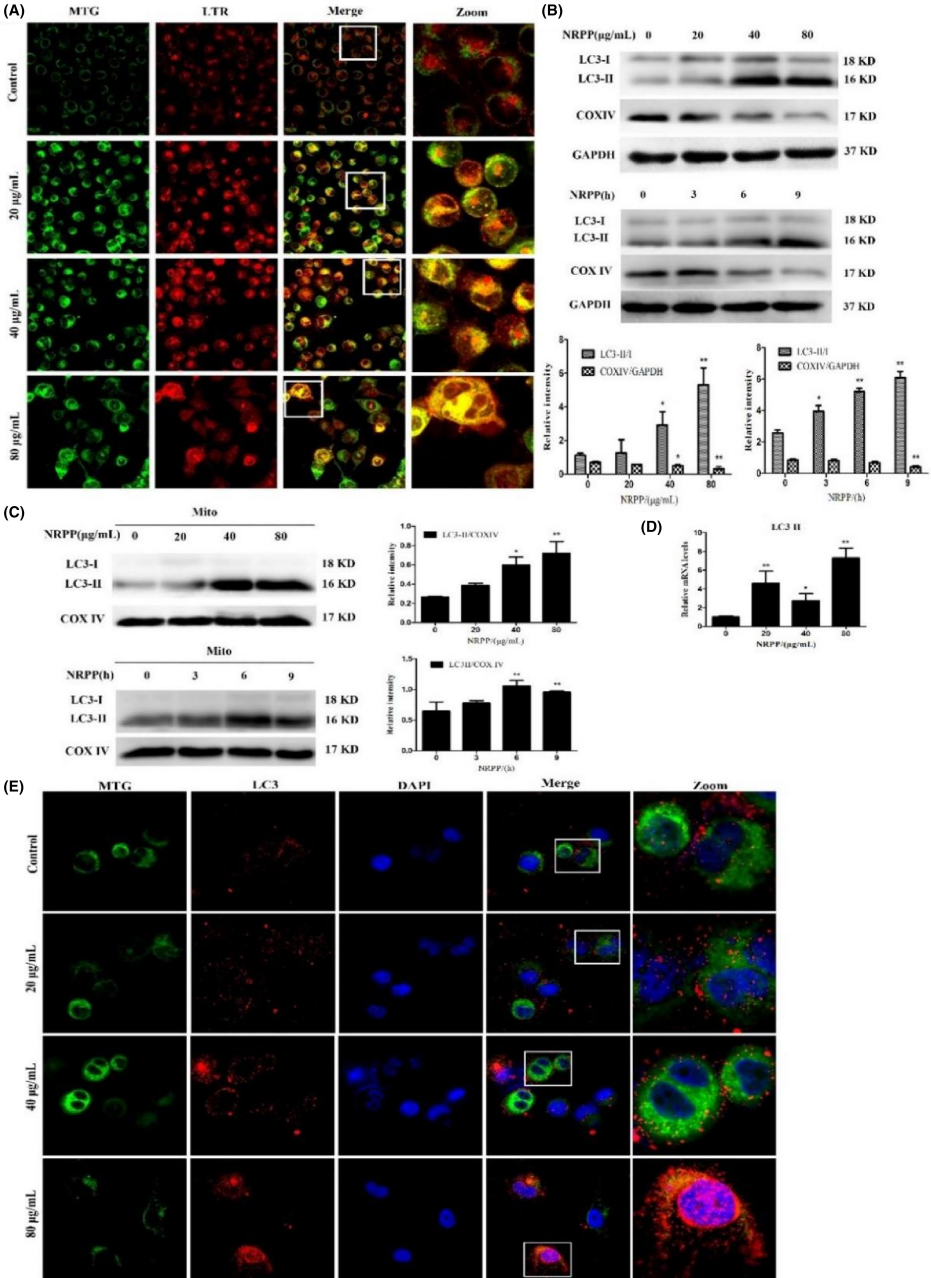
NRPP‐induced mitophagy in MDA‐MB‐435S cells. (A) The colocalization of mitochondria and lysosomes after NRPP exposure for 9 h. The images were obtained a using 60× objective. (B, C) The expression of LC3 and COXIV total proteins was measured by western blot. (C) The expression of LC3 in mitochondrial was tested by western blot, COXIV was the control. (D) The mRNA levels of LC3II were tested by quantitative RT‐PCR analysis after NRPP exposure for 9 h. (E) The immunofluorescence images were recorded after NRPP exposure for 9 h and cells were labelled with MTG, LC3 antibody and DAPI, respectively. The images were obtained by a laser confocal microscope using a 100× objective. *p < 0.05 and **p < 0.01 vs. control group.

To further explore whether NRPP could induce mitophagy in MDA‐MB‐435S cells, MTG and LTR staining was applied to research the colocalization of mitochondria and lysosomes. MTG is a green fluorescent probe that specifically labels the mitochondrion of living cells, and LTR is a red fluorescent probe that specifically labels lysosomes in living cells. The results displayed that with the raise of NRPP concentration, the mitochondrial green fluorescence and lysosome red fluorescence colocalized, and yellow fluorescence aggregation increased, indicating the occurrence of mitophagy (Figure 2A).

NRPP significantly increased the autophagy marker protein LC3II/I ratio and decreased the expression of the mitochondrial respiratory chain complex COXIV in a time‐ and dose‐relationship (Figure 2B). NRPP also increased mitochondrial LC3II (Figure 2C). Compared with the control, NRPP treatment dramatically improved the relative mRNA levels of LC3B (Figure 2D). Co‐immunofluorescence staining result suggested that the red fluorescence of LC3 was aggregated, and the numbers of yellow puncta and aggregates increased with NRPP treatment, especially at 80 μg/mL, indicating that the colocalization of LC3‐mitochondria was upregulated and mitophagy was enhanced. The co‐immunofluorescence images and mRNA analysis results further verified the conclusion of western blotting. Therefore, NRPP‐induced mitophagy in MDA‐MB‐435S cells.

### 3.3 NRPP‐induced mitophagy was regulated by P53/BNIP3/NIX pathway in MDA‐MB‐435S Cells

We explored the mechanism underlying NRPP‐induced mitophagy in MDA‐MB‐435S cells. The mitophagy signalling pathway mainly includes the PINK1/Parkin and mitophagy receptors, mainly including BNIP3, NIX, et al.^27^ Parkin and BNIP3 are transcription target p53 genes, as a tumour suppressor. p53 is also important for the occurrence and development of cancer.^28^ Therefore, the primary mitophagy proteins were tested in MDA‐MB‐435S treated with NRPP.

**FIGURE 3:**
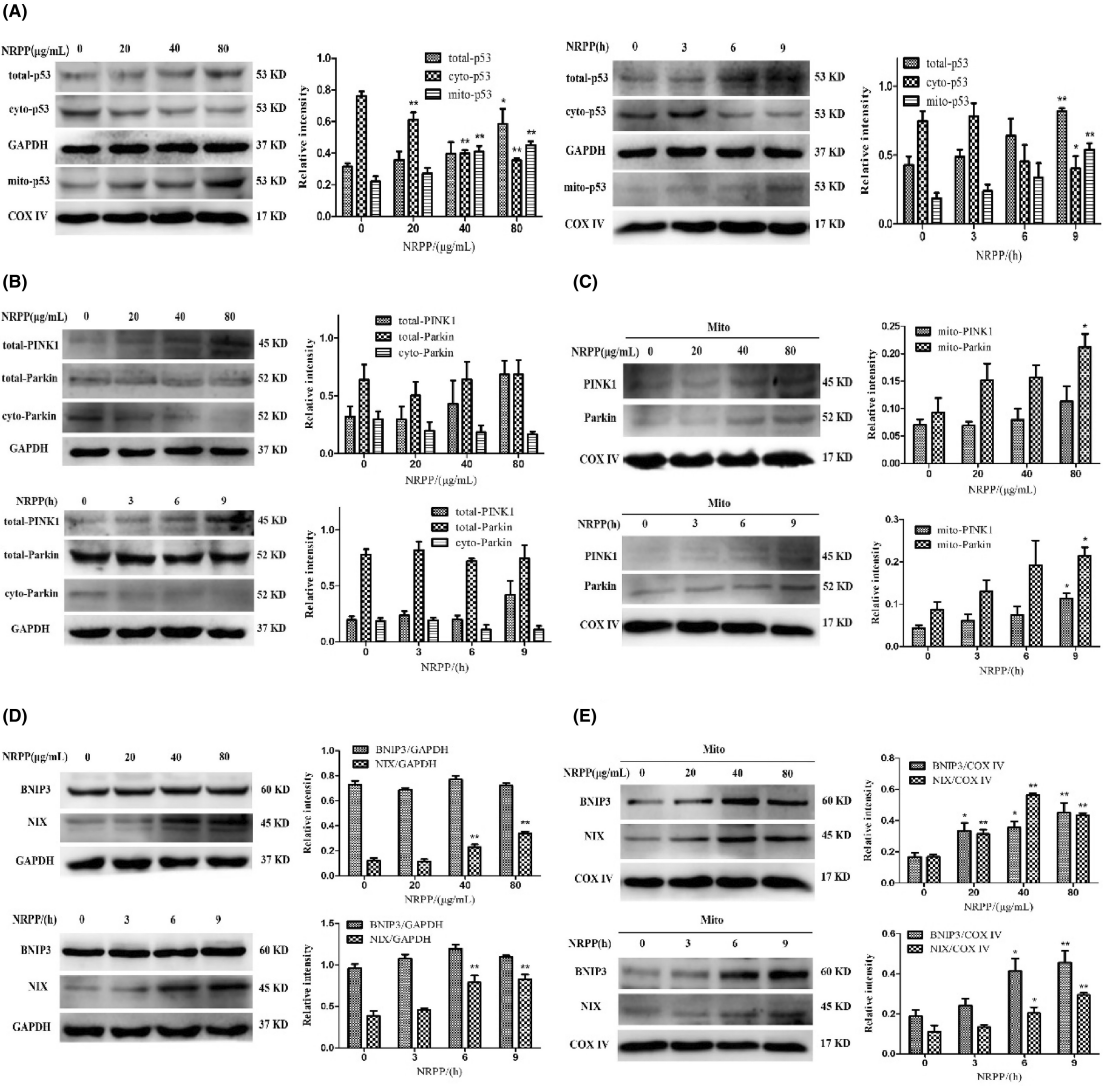
Effects of NRPP on mitophagy pathway proteins in MDA‐MB‐435S cells. The mitophagy pathway proteins were detected by western blot after NRPP‐treated with different concentrations and times. (A) The expression of total p53, cytosolic p53 and mitochondrial p53 proteins. (B, C) The expression of PINK1/Parkin pathway proteins. (D, E) The expression of BNIP3/NIX pathway proteins. GAPDH was regarded as a loading control for total and cytosolic proteins. COXIV was regarded as a loading control for mitochondrial protein. *p < 0.05 and **p < 0.01 vs. control group.

From Figure 3A, the findings hinted that NRPP markedly upgrades the total and mitochondrial levels of p53, but decreased the cytosolic level of p53 compared with those of the control, prompting that p53 might regulate NRPP‐induced mitophagy. Mitochondrial Parkin protein expression was remarkably increased by NRPP at 80 μg/mL. Mitochondrial PINK1 and Parkin proteins were significantly upregulated following NRPP‐treated for 9 h group (Figure 3C); while the total protein expressions of the PINK1 and Parkin were not affected from that on both times and doses aspects (Figure 3B), indicating that NRPP had no obvious effect on the PINK1/Parkin pathway. The BNIP3/NIX pathway results suggested that the total level of BNIP3 protein in cells showed no obvious change, but the total level of NIX protein increased significantly (Figure 3D), and the mitochondrial levels of BNIP3 and NIX proteins increased significantly (Figure 3E). In conclusion, NRPP may induce mitophagy via the p53/BNIP3/NIX pathway in MDA‐MB‐435S cells.

**FIGURE 4:**
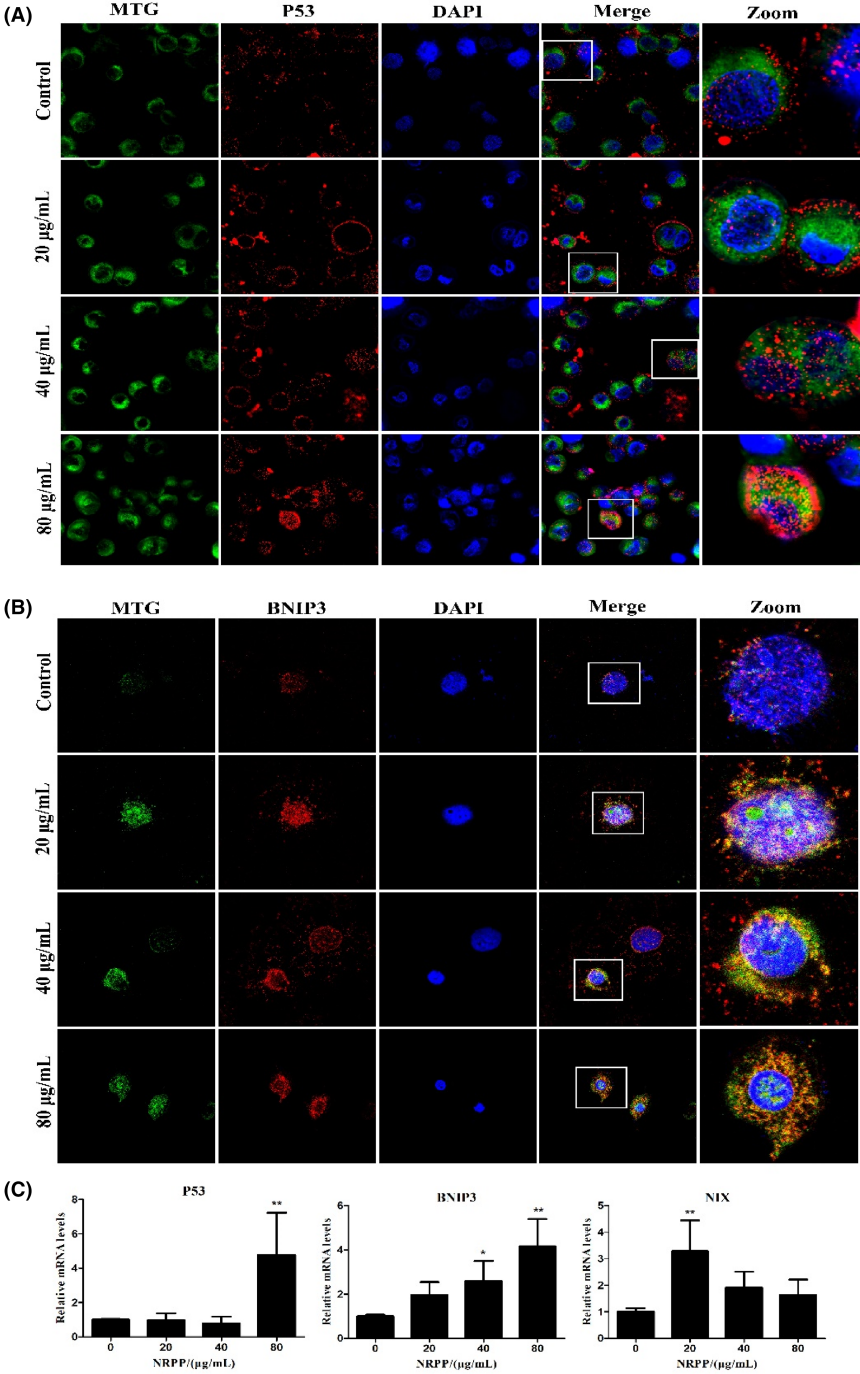
NRPP‐induced mitophagy in MDA‐MB‐435S cells through activating p53/BNIP3/NIX pathway. (A, B) The expression of p53 and BNIP3 and their colocalization with mitochondria were detected by immunofluorescence analyses. The immunofluorescence images of cells labelled with MTG, p53 or BNIP3 antibodies, and DAPI after NRPP exposure for 9 h. The representative images were obtained under a laser confocal microscope using a 100× objective. (C) The mRNA levels of p53, BNIP3 and NIX genes were measured by quantitative RT‐PCR analysis after NRPP exposure for 9 h. *p < 0.05 and **p < 0.01 vs. control group.

Next, we used co‐immunofluorescence analysis and RT‐PCR to verify this conclusion. Both p53 and BNIP3 levels of red fluorescence in MDA‐MB‐435S cells were markedly increased. Moreover, p53 and BNIP3 colocalized more with the mitochondria on NRPP‐treated in a dose‐effect relationship (Figure 4A,B). The mRNA expression of BNIP3 was gradually upregulated, p53 and NIX were higher than that in the control, but there was no concentration dependence (Figure 4C). The above quantitative and qualitative results further proved that NRPP induces mitophagy via the p53/BNIP3/NIX pathway.

### 3.4 NRPP inhibited tumour growth in MDA‐MB‐435S xenograft models

After confirming the inhibitory activity of NRPP in MDA‐MB‐435S cells by inducing mitophagy in vitro, we further established breast cancer xenograft models to validate the effect and its mechanism in vivo.

**FIGURE 5:**
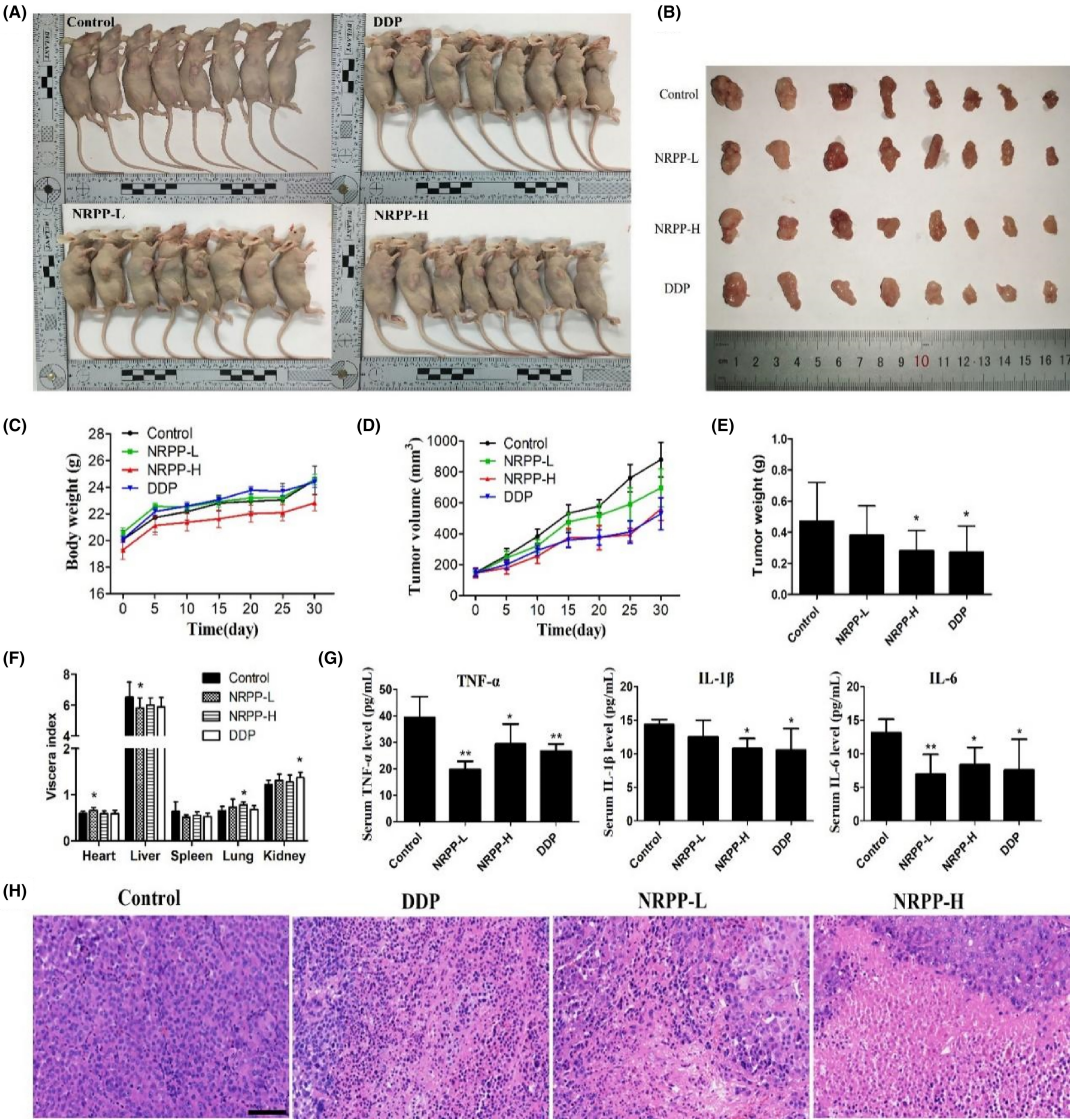
NRPP suppressed tumour growth in MDA‐MB‐435S xenograft mice. (A, B) Illustrations of mice and tumours. (C–E) The mice body weights, xenograft tumour volumes and xenograft tumour weight of mice in all groups (*n* = 8). (F) The viscera index of all groups (*n* = 8). (G) Serum inflammatory factor levels detected by ELISA Kit (*n* = 5). (H) Haematoxylin and eosin staining of tumours. Scale bar: 20 μm. *p < 0.05 and **p < 0.01 vs. control group.

During administration, the nude mice were in good mental condition. On Day 30, characteristic pictures of the mice and tumours were displayed in Figure 5A,B. The body weights in each group of mice presented a clear upward trend. The average weights of the mice treated with NRPP‐L or DDP were not obviously different from those of the control. The average weight of the NRPP‐H mice was slightly lighter than that of other groups, probably due to appetite loss (Figure 5C). In the last stage of treatment, the average volumes and weights of xenograft mice in other groups decreased compared with those of the controls (Figure 5D,E). The volume inhibition rates in the NRPP‐L, NRPP‐H and DDP groups were 20.76%, 36.21% and 39.84%, respectively. The weight inhibition rates in the NRPP‐L, NRPP‐H and DDP groups were 19.29%, 41.42% and 42.69%, respectively. The order of the antitumour efficacy of these three drug groups was DDP group > NRPP‐H group > NRPP‐L group.

There was no obvious change in the relative weights of organs, including the heart, liver, spleen, lung and kidney (Figure 5F). The ELISA data showed that the levels of TNF‐α, IL‐6 and IL‐1β in the NRPP‐L, NRPP‐H and DDP groups were all decreased, and there were certain significant differences (Figure 5G). These results revealed that the inhibition effect of NRPP on the growth of xenograft tumour might be related to the decrease in TNF‐α, IL‐6 and IL‐1β levels. Haematoxylin and eosin staining (Figure 5H) suggested that tumour cells were closely arranged, and no obvious necrotic areas were found in the control, while massive necrosis occurred in the treatment group.

### 3.5 Mechanism of NRPP‐induced mitophagy in MDA‐MB‐435S xenograft models

**FIGURE 6:**
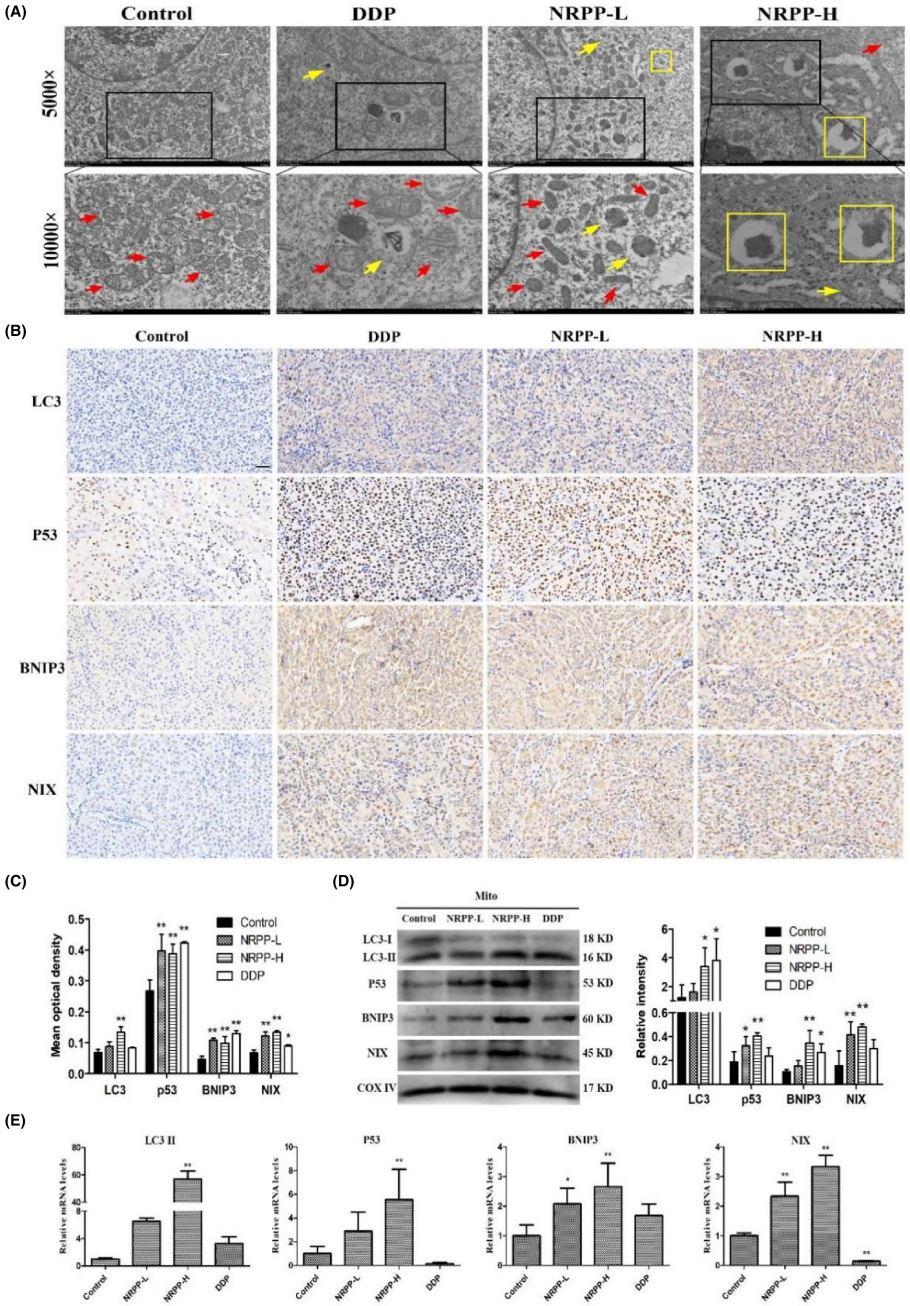
NRPP‐induced p53/BNIP3/NIX pathway‐mediated mitophagy in MDA‐MB‐435S xenograft nude mice. (A) The ultrastructures of the tumours were observed under TEM. The magnifications of the images were 5000X and 10,000X. Red arrows: mitochondria. yellow arrows: autophagosomes; yellow squares: autolysosomes. (B) IHC staining of LC3, p53, BNIP3 and NIX in tumour tissues. Scale bar: 20 μm. (C) The quantitative expression of positive optical densities in IHC staining using the Image‐Pro Plus software. (D) The mitochondrial protein levels of LC3, p53, BNIP3 and NIX in tumour tissues measured by western blot. COXIV was the control protein. (E) The mRNA levels of LC3II, p53, BNIP3 and NIX genes in tumour tissues measured by quantitative RT‐PCR analysis. *p < 0.05 and **p < 0.01 vs. control group.

In tumour‐bearing nude mice, we examined the ultrastructure of stripped tumour using TEM (Figure 6A). Many intact organelles, such as mitochondria, endoplasmic reticulum and ribosomes with normal morphology and structure were panic in the xenograft cells of the control. By contrast, in the treatment group found damaged mitochondrial membranes, broken cristae or disappeared. Also, vacuoles and autophagosomes were observed. Autolysosomes in the late stages of autophagy were observed in the NRPP‐L and NRPP‐H groups. This observation suggested that NRPP‐induced autophagy in xenograft tumour in vivo. Moreover, the increased optical density of LC3 observed by IHC staining also supported this conclusion (Figure 6B,C). Additionally, the markedly increased optical densities of p53, BNIP3 and NIX in tumour tissues treated with NRPP, as determined by IHC staining, supported the activation of mitophagy. Consistently, LC3, p53, BNIP3 and NIX proteins in the mitochondria of tumours (Figure 6D), as well as their mRNA levels (Figure 6E), were upregulated. To sum up, these results further demonstrated that NRPP can inhibit xenograft tumour growth via p53/BNIP3/NIX pathway‐mediated mitophagy initiation in MDA‐MB‐435S breast cancer xenograft models.

## 4 DISCUSSION

When cells are stimulated by hypoxia, nutritional deficiency or inflammation, intracellular reactive oxygen species increase, resulting in the pathological opening of mitochondrial permeability, the disappearance of cristae, swelling and depolarizing of the mitochondrial membrane and a reduction in MMP. This may lead to apoptosis and autophagy.^29^, ^30^ Rh‐123 staining showed that MMP decreased significantly with an increase in NRPP concentration, which is an early event of mitochondrial damage and might engender mitophagy. During the early stage of mitophagy, mitophagosomes form independently, recognizing and subsequently consumed mitochondria because of the unique double membranes, cristae of mitochondria and receptors on the outer membrane. At the final stage of mitophagy, mitolysosomes form with the fusion of mitophagosomes and lysosomes, followed by hydrolysis of cargoes via lysosomal enzymes.^31^, ^32^ MTG and LTR staining result showed that the colocalization of mitochondria and lysosomes was enhanced by NRPP in a concentration‐dependent manner, indicating mitophagy occurred. TEM observation of autophagosomes is considered the gold standard for autophagy research,^31^ so we observed damaged mitochondria and mitophagosomes using it. These results indicate that NRPP may induce mitophagy in MDA‐MB‐435S cells.

Nevertheless, the function of autophagy in cancer treatment remains controversial. Some researchers believe that autophagy could also promote tumour growth.^33^ Mitophagy is a selective process. Normal mitophagy can be used as a preventive mechanism and could degrade damaged mitochondria, whereas excessive mitophagy can cause abnormal mitochondrial circulation and energy metabolism disorders, and induce cell death.^34^ Mitophagy is a complicated process that causes the abnormal expression of various proteins. Our research indicated that NRPP could increase the expression of total LC3II/I and mitochondrial LC3II in a concentration‐ and time‐dependent manner while downregulating COXIV expression in MDA‐MB‐435S cells. Immunofluorescence results hinted that NRPP upregulated mitochondrial LC3, which was agreed with the western blot results. RT‐PCR data suggested that the LC3B mRNA level increased dramatically compared with that in the control. These findings confirmed that mitochondria were damaged and mitophagy was initiated after NRPP treatment.

PINK1 is a serine–threonine kinase that participated in regulating calcium accumulation at an incipient stage of mitophagy, depolarizing mitochondrial membrane and removing depolarized mitochondria in the late stage.^34^ The PINK1/Parkin pathway is currently a hotspot and was identified in breast cancer.^35^, ^36^ Our findings showed that NRPP did not have a splendid impact on the PINK1/Parkin pathway in MDA‐MB‐435S cells, implying that NRPP‐induced mitophagy may be mediated by other pathways.

Except for the typical PINK1/Parkin pathway, BNIP3, NIX and other receptor pathways can mediate mitophagy, inhibiting many cancers. BNIP3 and NIX are attached to the outer membranes of mitochondria and contain LIR domain. LIR domains can directly combine with LC3 to activate mitophagy and highly expressed in breast ductal carcinoma in situ.^37^, ^38^ Deletion of BNIP3 is a prognostic indicator of triple‐negative breast cancer metastasis. Mitochondrial dysfunction caused by mitophagy imperfections can promote tumour development, providing a new approach for breast cancer treatment.^39^ In our study, western blot results suggested that NRPP upregulated the expression of BNIP3 and NIX in mitochondria, which was further verified via immunofluorescence and RT‐PCR, indicating that BNIP3/NIX pathway‐mediated NRPP‐induced mitophagy in MDA‐MB‐435S cells.

As a cellular sensor, the p53 suppressor is essential to DNA damage and repair, hypoxia, oxidative and nutritional stress, cell cycle, necrosis, apoptosis and autophagy in the cytosol and mitochondria.^40^, ^41^ Studies have shown that Parkin is a p53 target gene. Cytosolic p53 can bind to Parkin, hindering its translocation to injure mitochondria, thus blocking mitophagy.^42^ Similarly, BNIP3 and NIX are transcriptionally regulated by p53. p53 can inhibit BNIP3 levels by combining with the p53‐response element and looking for the corepressor for the BNIP3 promoter.^43^ In radiation‐resistant cancer cells with p53 deletions, oxygen consumption decreased, glycolysis increased, and damaged mitochondria were observed. By contrast, p53/BNIP3‐regulated mitophagy overcame chronic radiation exposure, suggesting that targeting p53 and mitophagy may be a potential target in cancer therapy.^44^ However, the specific interaction that p53 regulates BNIP3 and NIX is complex and not yet clear. Our researches indicated that NRPP upregulated the expression of total and mitochondrial p53 proteins, but downregulated the cytosolic p53 protein, and accelerated p53 translocation to the mitochondria, suggesting that NRPP‐induced mitophagy may be concerned in the regulation of p53.

To evaluate the efficacy and mechanism of NRPP in breast cancer deeply, we constructed a breast cancer xenograft model. We found NRPP excellently inhibited tumour growth and had no obvious toxic side effect on mice weight and viscera index. These results preliminarily indicated that NRPP was relatively safe and had no obvious toxicity to nude mice within the dose range used in this experiment. However, its toxicity should be further confirmed by examining the indices of liver and kidney function and pathological changes. Moreover, the results of TEM, IHC, western blotting and RT‐PCR analysis also confirmed that NRPP treatment induced mitophagy via the p53/BNIP3/NIX signalling pathway. In our subsequent research, we will use a mitophagy inhibitor/activator and knock down mitophagy pathway receptors to further verify our conclusions. Nevertheless, the antitumour mechanism of NRPP in vivo is complex, and many possible mechanisms need to be further explored to find sufficient evidence for NRPP in breast cancer therapy.

**FIGURE 7:**
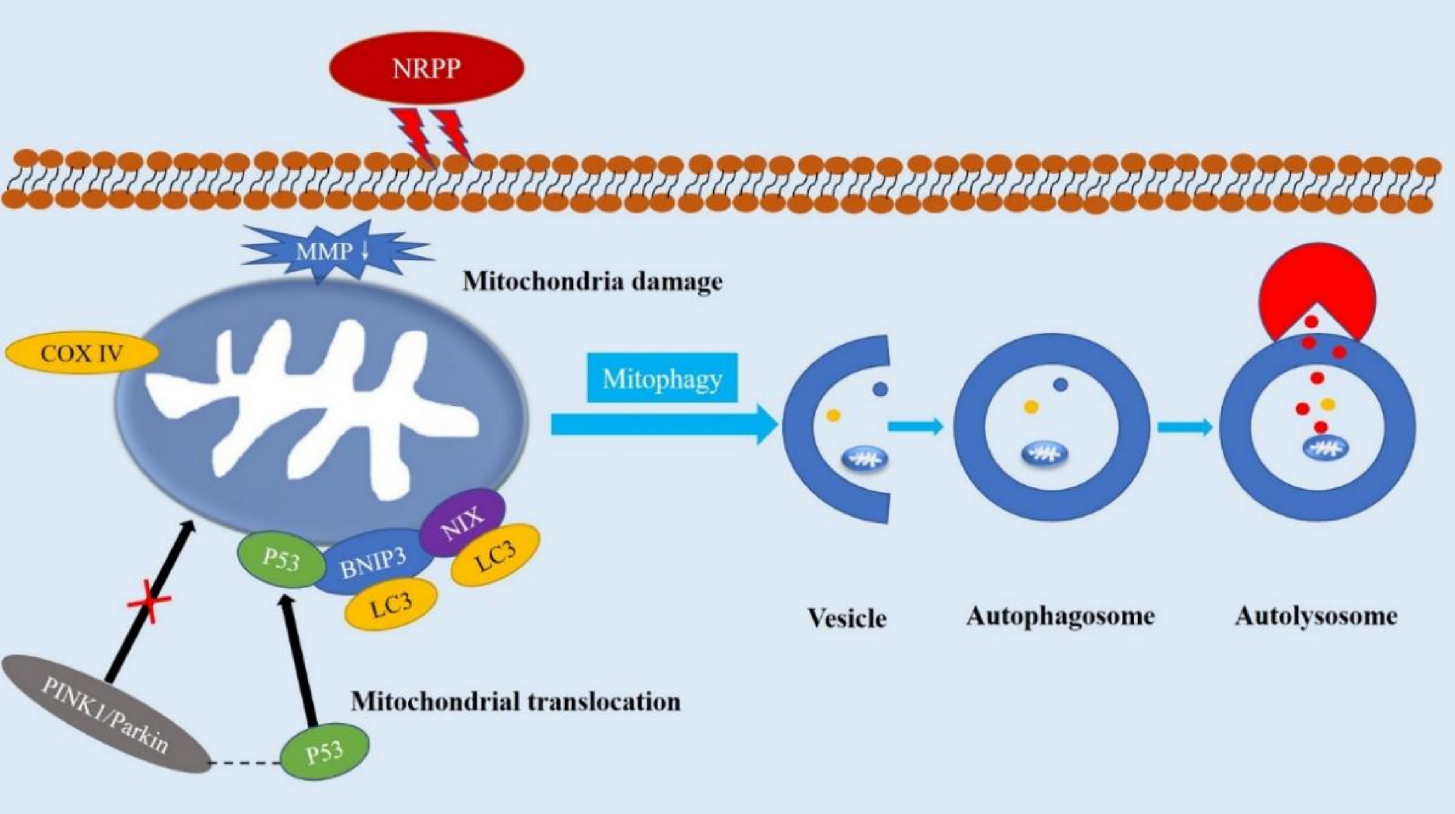
Schematic diagram depicting the molecular mechanism of NRPP against breast cancer through mitophagy.

In summary, this research found that NRPP could suppress breast cancer MDA‐MB‐435S cell proliferation and tumour growth via mitophagy. Moreover, we found that NRPP‐induced mitophagy was mediated by the p53/BNIP3/NIX signalling pathway (Figure 7), providing a novel pathway and target for NRPP in the treatment of breast cancer. Therefore, NRPP could be a prospective anti‐breast cancer agent that targets mitophagy.

## AUTHOR CONTRIBUTIONS

**Xiaoli Yang:** Formal analysis (equal). **Yi Liu:** Formal analysis (equal). **Xiuqiao Zhang:** Methodology (lead); project administration (lead); writing – review and editing (lead). **Jiahui Fang:** Data curation (equal); formal analysis (lead); writing – original draft (lead). **Juan Xi:** Formal analysis (equal). **Ling Gong:** Formal analysis (equal). **Xue Zou:** Formal analysis (equal). **Chun Gui:** Funding acquisition (lead); methodology (lead); writing – review and editing (equal).

## FUNDING INFORMATION

This research was supported by the Scientific Research Foundation of Education Department of Hubei Province in China (No. Q20202004) and the Traditional Chinese Medicine high‐level key discipline construction project of National Administration of Traditional Chinese Medicine (2023)‐Medicinal Mineralogy.

## CONFLICT OF INTEREST STATEMENT

The authors declare that they have no known competing financial interests or personal relationships that could have influenced the work reported in this study.

## Data Availability

The datasets generated for this study can be accessed upon request by the corresponding author.
